# Place attachment mediates links between pro-environmental attitudes and behaviors among visitors to Mt. Bukhan National Park, South Korea

**DOI:** 10.3389/fpsyg.2024.1338650

**Published:** 2024-02-13

**Authors:** Jee In Yoon, KangJae “Jerry” Lee, Lincoln R. Larson

**Affiliations:** ^1^Department of Coaching, Kyung Hee University, Seoul, Republic of Korea; ^2^Department of Parks, Recreation, and Tourism, University of Utah, Salt Lake City, UT, United States; ^3^Department of Parks, Recreation, and Tourism Management, North Carolina State University, Raleigh, NC, United States

**Keywords:** place attachment, environmental behavior, environmental attitude, sense of place, environmental stewardship

## Abstract

**Introduction:**

Efforts to understand visitors’ participation in pro-environmental behaviors (PEB) are important for protected area management. Previous research in nature-based recreation settings suggests environmental attitudes may affect PEB, and that these relationships might be mediated by different dimensions of place attachment (place identity and place dependence).

**Methods:**

We used structural equation modeling to test the mediating effect of hikers’ place attachment in the relationship between environmental attitudes and PEBs that occur within (on-site) and outside a protected area (off-site): Mt. Bukhan National Park in South Korea.

**Results:**

Results showed that cognitive (environmental knowledge) and affective (environmental sensitivity) components of environmental attitudes were significant predictors for place attachment. Place identity was linked to off-site PEB, while place dependence was a key antecedent for both off-site and on-site PEBs.

**Discussion:**

Our findings could help researchers and practitioners better understand how place attachment forms and how it can impact outdoor recreationists’ behavior, ultimately helping to promote PEBs and facilitate sustainable management goals.

## 1 Introduction

Due to continuing anthropogenic threats such as global climate change, rising urbanization, and overcrowding in many outdoor recreation settings, understanding human behavior is an important aspect of park and natural resource management (Orishimo, 2012; Rommel et al., 2015; Bradley et al., 2020). Moreover, many park managers and practitioners are charged with a so-called “dual mandate” (Higham et al., 2016)—they must protect the natural environment while providing positive visitor experiences. For all of these reasons, predicting and -promoting the PEBs of outdoor recreationists is a critical issue in parks and natural resource management.

Many researchers view environmental attitudes as the key to understanding environmental behavior (Heberlein, 2012; Liu et al., 2020). Environmental attitudes are often defined as a person’s positive or negative evaluation of the natural environment (Kaiser et al., 1999). To date, researchers have studied various forms of environmental attitudes, such as environmental concern (Weigel, 1983; Takala, 1991; Fransson and Gärling, 1999), attitude toward nature (Becker et al., 1981; Van der Pligt et al., 1986; Grob, 1995), and environmental values (Karp, 1996; Lee et al., 2014), often merging the concept of environmental attitudes into larger worldviews such as the New Ecological Paradigm (NEP; Dunlap et al., 2000). However, most previous studies have considered either cognitive or affective aspects of environmental attitudes separately, and not both simultaneously (Manfredo et al., 1992; Quoquab and Mohammad, 2020). For example, environmental knowledge (Simmons and Widmar, 1990; Maurer and Bogner, 2020), a cognitive component, and environmental sensitivity (Perterson, 1982; Canosa et al., 2020), an affective component, could be accounted for concurrently to represent a more comprehensive portrayal of environmental attitudes (Cheng and Wu, 2015; Quoquab and Mohammad, 2020). Studies focused on tourism (Huang and Shih, 2009; Cheng and Wu, 2015) and environmental psychology (Duerden and Witt, 2010; Priadi et al., 2018) have shown that these two components of attitudes may have a significant influence on PEBs. Yet, to date, little research has examined how cognitive and affective aspects of environmental attitudes interact to influence environmental behavior in a nature-based recreation setting (Liu et al., 2020).

Although numerous studies have revealed significant links between pro-environmental attitudes and behavior (Bamberg and Möser, 2007; Kaiser et al., 2007; Ritter et al., 2015), more recent reviews have revealed a complex relationship between these concepts with a strength of association that varies based on contextual factors such as normative influences or perceived behavioral control (Ajzen, 2012; Heberlein, 2012). Place attachment is another contextual factor of potential significance, yet few studies have investigated the mediating effect of place attachment on the relationship between pro-environmental attitudes and behaviors (e.g., Ramkissoon et al., 2012). Environmental attitudes can affect place attachment (Budruk et al., 2009; Brehm et al., 2013), which can in turn affect PEB (Vaske and Kobrin, 2001; Halpenny, 2010; Larson et al., 2018b; Song and Soopramanien, 2019). But few previous studies have empirically tested this mediation effect in outdoor recreation settings. Cheng and Wu’s (2015) study of visitors to the Penghu Islands in Taiwan is one notable exception. They found that the environmental knowledge and environmental sensitivity of recreationists positively influenced their place attachment, which in turn predicted general and specific PEBs. However, their study did not examine the differential effect of place identity and place dependence, two key dimensions of place attachment, on PEB.

### 1.1 Multiple dimensions of PEB

Pro-environmental behavior (PEB) is a multidimensional construct delineating actions that benefit the natural environment, enhance environmental quality, or minimize harm to the environment (Steg and Vlek, 2009). For instance, Larson et al.’s (2015) study on rural residents of New York, United States, identified various PEB domains such as conservation lifestyle, environmental citizenship, and land stewardship. Furthermore, in the study of Taiwanese island tourists’ intention to engage in environmentally responsible behaviors, Cheng and Wu (2015) considered an environmentally responsible behavior from two dimensions: general behavior that occurs outside the recreational site (off-site) such as reading books about environmental issues related to a particular area, and specific behavior is actions happening inside the site (on-site) such as participating in activities to clean the area. Similarly, Lange and Dewitte (2019) measured PEB in two similar categories, domain-general and domain-specific behaviors, and Larson et al. (2018b) conceptualized PEB in two dimensions, low-effort (mostly on-site) and high-effort (mostly off-site) behavior. In each case, researchers distinguished between behaviors that occur in a park and might have a direct impact on park resources and management priorities, and behaviors that occurred outside of a park that might have a broader impact on global conservation efforts. In our study, we mirrored this approach and operationalized PEB as a two-dimensional construct (on-site and off-site), to examine visitors’ intent to participate in PEBs within the boundaries of the park itself and out in their broader lives.

### 1.2 Components of environmental attitudes: environmental knowledge and sensitivity

Environmental knowledge is a cognitive component of environmental attitudes which refers to an individual’s level of understanding about the environment and human impacts on the environment (Ramsey and Rickson, 1976; Simmons and Widmar, 1990). Several researchers have noted that environmental knowledge is a critical precursor to PEB. For example, Ramdas and Mohamed (2014) discovered that people who possessed elevated environmental knowledge were more likely to purchase green products. Similarly, Flamm (2009) noted a positive relationship between consumers’ environmental knowledge and PEBs. In addition, Lawhon et al. (2017) documented that self-reported environmental knowledge influenced the behavioral intention of engaging in leave-no-trace actions in three U.S. state parks. In their study of Chinese residents, Liu et al. (2020) noted an association between environmental knowledge and PEB, but this relationship was indirect and mediated by a variety of other factors.

Environmental sensitivity is an affective aspect of environmental attitudes that refers to “a set of affective attributes which result in an individual viewing the environment from an empathetic perspective” (Perterson, 1982, p. 5). Although environmental sensitivity is a fundamental element of environmental citizenship and PEB that has been emphasized in the environmental education literature (Hungerford and Volk, 1990; Chawla, 1998), the concept has received less attention. However, in a nature-based tourism context, Cheng and Wu (2015) found that the strongest predictors of PEB were environmental sensitivity and place attachment. Bustam et al.’s (2003) also studied the influence of environmental sensitivity on recreation site preference. Other studies have broadly explored links between related concepts, such as environmental concern and outdoor recreation participation, often yielding mixed results (Berns and Simpson, 2009; Ghazvini et al., 2020). Ultimately, although few researchers have focused explicitly on environmental knowledge and sensitivity and precursors to PEB, existing research suggests that both concepts could significantly influence the PEB of park visitors and other outdoor recreationists.

### 1.3 Role of place attachment in the attitude-behavior relationship

Place attachment might have a significant impact on PEB within (on-site) and outside of (off-site) the park area (Vaske and Kobrin, 2001; Halpenny, 2010). Place attachment refers to the emotional bond between humans and environment (Low and Altman, 1992). Existing literature supports the idea that place attachment is a multidimensional construct (Kyle et al., 2005; Tumanan and Lansangan, 2012). Two of the most commonly-described and widely recognized dimensions of place attachment are place identity (one’s identity expressed within the physical environment; Proshansky, 1978) and place dependence (the functional utility of a setting in satisfying one’s goal; Stokols and Shumaker, 1981).

Some research has demonstrated links between environmental attitudes and place attachment, especially among outdoor recreation enthusiasts (Brehm et al., 2006; Budruk et al., 2009). Place attachment may be particularly important in nature-based recreation contexts where quality experiences depend on the resource itself (Larson et al., 2018a). Jorgensen and Stedman’s (2006) investigation of predictors of sense of place documented that lakeshore property owners’ perceptions on environmental features (e.g., native vegetation and shoreline development) were the strongest predictors of their place identity, attachment, and dependence. Place attachment can also be associated with recreationists’ perceptions regarding setting conditions (Kyle G. et al., 2004) and the impacts of recreation activities on the environment (White et al., 2008; Eder and Arnberger, 2012). Thus, place attachment can influence the way recreationists think about a natural resource and impacts to that resource, potentially influencing their intent to engage in behaviors that protect the resource.

Many researchers have studied the relationship between place attachment and PEB (Stedman, 2002; Masterson et al., 2017; Larson et al., 2018a). Høyem (2020) found that developing a meaningful connection with natural environment was crucial in terms of increasing PEBs. Similarly, Clayton (2003) found that people who strongly identified with the natural environment demonstrated higher engagement in environmentally sustainable actions than individuals who displayed a weaker identification. Kaiser and Wilson (2000) also showed that attachment to a natural environment significantly predicted general ecological behavior such as ecological garbage removal, water/power conservation, and ecological automobile use when controlling for residential area, length of residence, education, age, and gender. Furthermore, Halpenny (2010) found that place attachment to national parks in Canada positively predicted both place-specific (on-site) and general (off-site) pro-environmental behavioral intentions.

**Figure 1:**
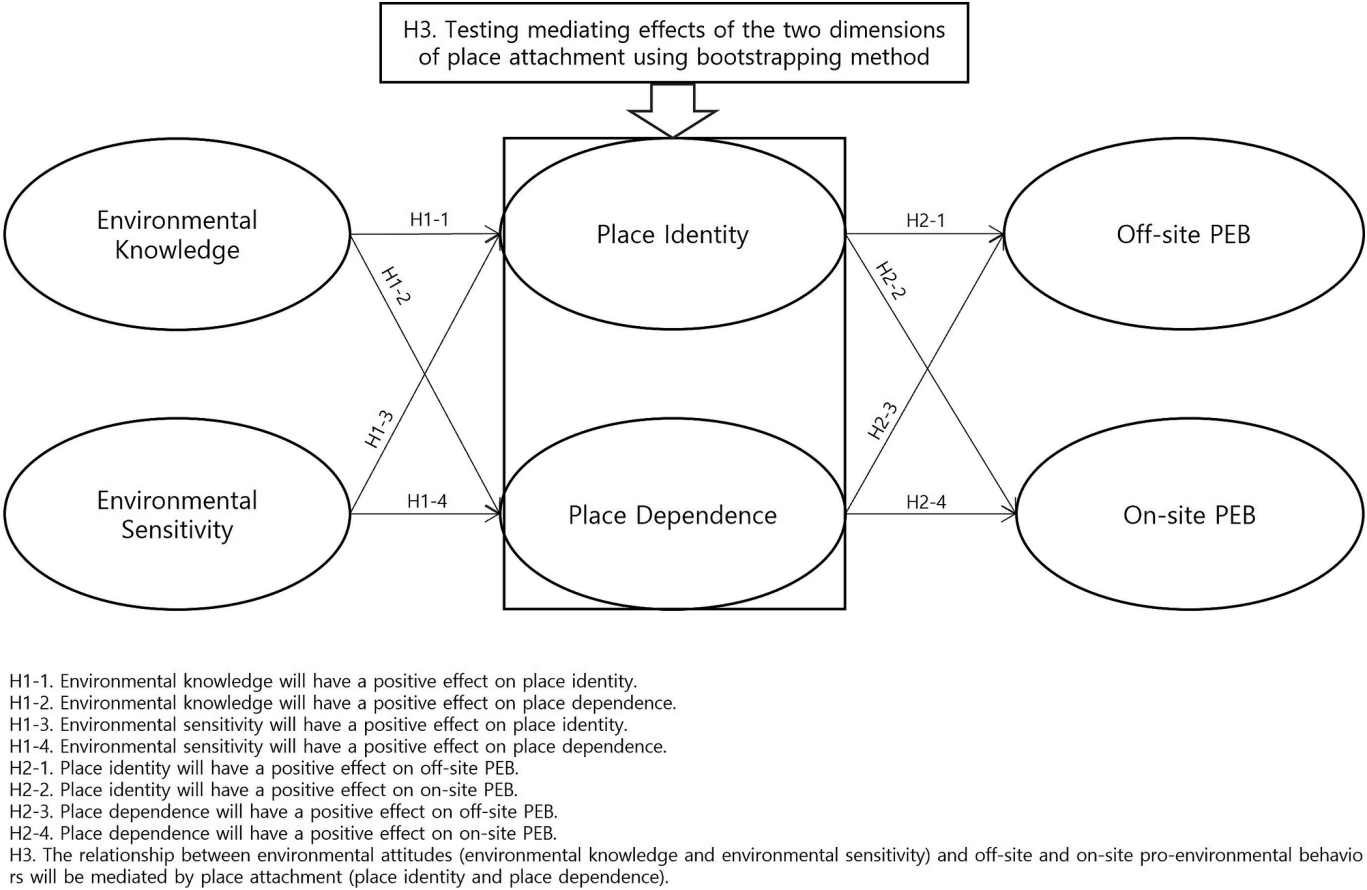
Hypothesized model of relationships among multiple dimensions of environmental attitudes, place attachment, and pro-environmental behavior.

Despite the wealth of research on these topics, few studies have simultaneously considered associations among PEB, cognitive and affective components of environmental attitudes, and multiple dimensions of place attachment in an outdoor recreation setting. To expand this growing body of research, we specifically tested whether the two dimensions of place attachment (i.e., place identity and place dependence) mediated the relationship between the two types of environmental attitudes (i.e., environmental knowledge and environmental sensitivity) and off-site and on-site PEBs in a popular Korean national park. As illustrated in Figure 1, we tested the following hypotheses:

*H1*: Environmental attitudes (environmental knowledge and environmental sensitivity) will have a positive effect on place attachment (place identity and place dependence).

*H1-1*: Environmental knowledge will have a positive effect on place identity.

*H1-2*: Environmental knowledge will have a positive effect on place dependence.

*H1-3*: Environmental sensitivity will have a positive effect on place identity.

*H1-4*: Environmental sensitivity will have a positive effect on place dependence.

*H2*: Place attachment (place identity and place dependence) will a have positive effect on off-site and on-site PEB.

*H2-1*: Place identity will have a positive effect on off-site PEB.

*H2-2*: Place identity will have a positive effect on on-site PEB.

*H2-3*: Place dependence will have a positive effect on off-site PEB.

*H2-4*: Place dependence will have a positive effect on on-site PEB.

*H3*: The relationship between environmental attitudes (environmental knowledge and environmental sensitivity) and off-site and on-site pro-environmental behaviors will be mediated by place attachment (place identity and place dependence).

## 2 Methods

### 2.1 Study context and data collection

National parks are most visited outdoor recreation sites in South Korea and contain natural environmental of national significance (Oh and Kim, 2013). To date, outdoor recreation research has been mainly conducted in the Western countries, often excluding East Asian context (Walker et al., 2001). Bukhansan Dulle-gil, one of the most visited outdoor recreation sites in South Korea, was selected to address this research gap.

**Figure 2:**
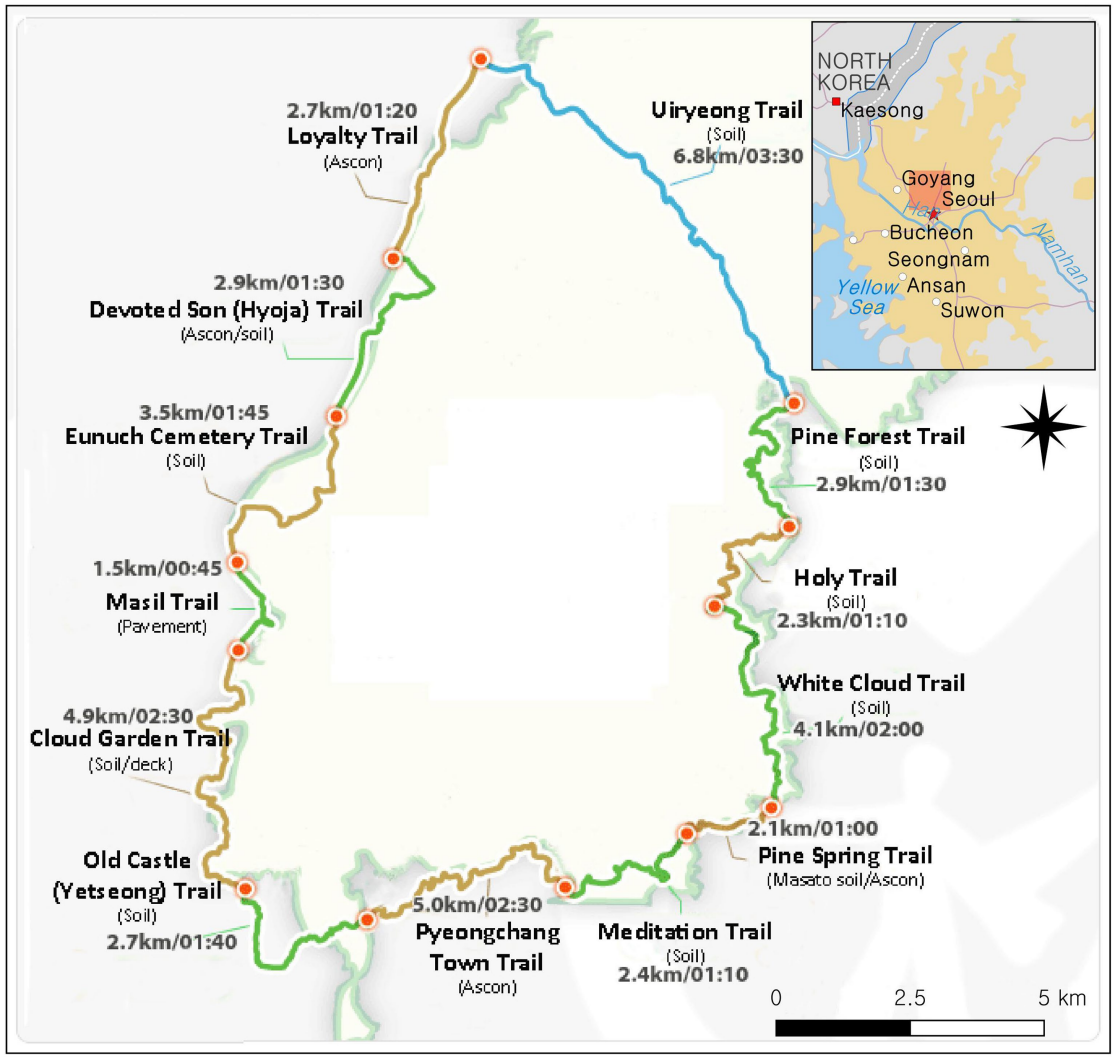
The map of Bukhansan National Park Dulle-gil and nearby hiking trails.

Mt. Bukhan Dullegil (see Figure 2) is a hiking trail along the foothills of Mt. Bukhan National Park in the northern part of Seoul, South Korea. In total the trail spans 71.8 km, comprising the Mt. Bukhan section (45.7 km) opened in September 2010 and the Mt. Dobong section (26.1 km) opened in June 2011. The number of visitors to Mt. Bukhan National Park is approximately 5.5 million per year (Korean Statistical Information Service, 2020). The park is located near the center of Seoul and is easily accessible by public transport, making it one of the most popular and most heavily impacted national parks in the country (Seo and Ko, 2013). The unique natural environment and popularity of the park makes it an ideal research context to understand the relationship between environmental attitude, place attachment, and PEBs.

Six research assistants conducted intercept surveys at four trails on the study site: Uiryeong Trail, Pine Forest Trail, Old castle (Yetseong) Trail, and Devoted Son (Hyoja) Trail. These four locations represent the park’s main highlights and attract the most visitors throughout the year (see Table 1). The data were collected during the second weekend of October 2018, one of the peak seasons. Research assistants randomly approached potential respondents at the entrance/exit points of the hiking trails. In each encounter with a visitor, the research assistants first explained the purpose of the survey and requested voluntary participation. A total of 550 copies of the questionnaire were distributed to potential respondents, and 432 copies were received by the assistants (response rate: 78.56%). After excluding 29 surveys that contained incomplete/insincere responses, 403 surveys were used in the final analysis.

**Table 1:** The characteristics of trails at Mt. Bukhan Dullegil, South Korea, where study sampling occurred in 2018.

Trail	Km	Surface	Surroundings	Theme
Pine Forest Trail^*^	2.9	Soil	Tree	Forest/recreation
Holy Trail	2.3	Soil	Tree	History/culture
White Cloud Trail	4.1	Soil	Tree	Forest/recreation
Pine Spring Trail	2.1	Masato soil/Ascon	Tree/building	Leisure/culture
Mediation Trail	2.4	Soil	Tree	Nature/exploration
Pyeongchang Town Trail	5.0	Ascon	Building	Landscape/viewpoint
Old Castle (Yetseong) Trail^*^	2.7	Soil	Tree	Landscape/viewpoint
Cloud Garden Trail	4.9	Soil/deck	Tree	Landscape/viewpoint
Masil Trail	1.5	Pavement	Tree/building	History/culture
Eunuch Cemetery Trail	3.5	Soil	Tree	Landscape/viewpoint
Devoted Son (Hyoja) Trail^*^	2.9	Ascon/soil	Road	Countryside/leisure
Loyalty Trail	2.7	Ascon	Road	Countryside/leisure
Uiryeong Trail^*^	6.8	Soil	Tree	Nature/exploration

*Trails selected for our data collection based on the number of visitors per trail and the discussion with Mt. Bukhan National Park Service.

### 2.2 Measurement of constructs

The specific questions we used for measuring each construct are reported in Table 2. We measured environmental knowledge using four items from the scale developed by Haron et al. (2005) to assess individuals’ understanding of environmental problems stemming from recreation behavior (e.g., I know that excessive recreational activities will damage the natural environment of the site). We measured environmental sensitivity using four items adapted from Sivek’s (2002) scale. The four items captured general affection toward the natural environment (e.g., I appreciate the natural environment of Mt. Bukhan Dullegil) as well as specific affective responses to the study site (e.g., I care about the natural environment of the site). We measured place attachment with the scale originally developed by Williams and Roggenbuck (1989) and later adopted by outdoor recreation researchers (Kyle G. T. et al., 2004). Four items were used to capture place identity (e.g., I have a special feeling for the trail) and another four items measured place dependence (e.g., It is more important to visit this trail than other similar destinations). We measured PEB with six items used in Smith-Sebasto and D’Costa’s (1995) study of the university students’ PEB. These consisted of four items about off-site PEB that might occur outside park boundaries in people’s broader lives (e.g., I discuss the environmental protection of Mt. Bukhan Dullegil with others) and two items about on-site PEB that might occur at the park itself (e.g., I pick up trash and branches when I see them on the trail). All items were rated on a 5-point Likert-type scale (1 = *strongly disagree* - 5 = *strongly agree*). Additionally, the survey included questions about the respondents’ sociodemographic information such as age, gender, education level, and place of residence. Table 2 lists all of the items and their corresponding dimensions.

**Table 2:** Results of confirmatory factor analysis examining different dimensions of environmental attitudes, place attachment, and pro-environmental behavior among visitors to Mt. Bukhan National Park, South Korea (*n* = 403).

Dimension/item	Mean (SD)	λ	CR	AVE	α
Environmental knowledge			0.840	0.570	0.827
I know that excessive recreational activities will damage the environments of the mountain	3.937 (1.061)	0.690			
I know that carbon dioxide emissions by automobiles and motorcycles will pollute the mountain	4.181 (1.003)	0.807			
I know that over extensive tourism development will sacrifice natural resources and environments	4.294 (0.901)	0.816			
I know that, in the trip, the use of green tableware, such as bowls and chopsticks will avoid damage to the environment	4.297 (0.944)	0.700			
Environmental sensitivity			0.864	0.619	0.860
I enjoy natural environments	4.121 (1.029)	0.842			
I am interested in the ecological preservation in Mt. Bukhan Dullegil	3.995 (1.022)	0.926			
I appreciate the natural environment of Mt. Bukhan Dullegil	3.907 (1.057)	0.702			
I care about the impact of my living habits on the natural environments of Mt. Bukhan Dullegil	3.793 (1.117)	0.648			
Place identity			0.913	0.724	0.925
Visiting Mt. Bukhan Dullegil has a deep meaning for me	3.816 (0.993)	0.774			
I have a strong sense of identifying with Mt. Bukhan Dullegil	3.563 (1.068)	0.831			
I have a strong sense of belonging in regard to Mt. Bukhan Dullegil	3.568 (1.066)	0.920			
I have a special feeling for Mt. Bukhan Dullegil	3.579 (1.008)	0.874			
Place dependence			0.935	0.783	0.934
I enjoy traveling in Mt. Bukhan Dullegil more than other hiking destinations	3.695 (1.037)	0.880			
I am more satisfied with visiting Mt. Bukhan Dullegil than other hiking destinations	3.740 (1.040)	0.924			
It is more important to visit Mt. Bukhan Dullegil than other hiking destinations	3.682 (1.054)	0.904			
No other locations can replace the hiking of Mt. Bukhan Dullegil	3.453 (1.128)	0.829			
Off-site PEB			0.902	0.698	0.895
I try to solve the environmental problems in Mt. Bukhan Dullegil	3.603 (0.959)	0.812			
I read the reports, advertising, and books related to the environments of Mt. Bukhan Dullegil	3.468 (1.034)	0.785			
I discuss with others about environmental protection of Mt. Bukhan Dullegil	3.350 (1.058)	0.883			
I try to convince companions to adopt positive behaviors in the natural environments of Mt. Bukhan Dullegil	3.407 (1.076)	0.859			
On-site PEB			0.852	0.743	0.845
I pick up trash and branches when I see them in the mountain	3.746 (0.954)	0.846			
I participate in activities to clean the mountain (such as participating in an event for picking up trash in the mountain)	3.695 (1.033)	0.878			

CFA fit indices: χ^2^ = 1154.942, df = 205, RMSEA = 0.080, CFI = 0.950, NNFI = 0.955.

### 2.3 Data analysis

We tested a hypothesized model (Figure 1) using a two-step approach for latent variable modeling commonly employed in environmental psychology research (Anderson and Gerbing, 1988; Zhang et al., 2018). First, we used confirmatory factor analysis (CFA) to validate the theorized factor structure of our hypothesized model for all six constructs (two dimensions each for environmental attitudes, place attachment, and PEB). We assessed internal consistency using Cronbach’s alpha coefficients, aiming for values greater than 0.80 (Hair et al., 2009). Our threshold for acceptable factor loadings was 0.60, exceeding the standard suggested by previous studies (Bagozzi and Yi, 1988; Brown and Moore, 2012). We also evaluated the composite reliability (CR) of all dimensions and the average variance extracted (AVE), aiming for values above 0.80 and 0.50, respectively (Fornell and Larcker, 1981; Sahoo, 2019).

Variables in the hypothesized model were assessed for normality by examining skewness and kurtosis. Especially in studies with samples greater than 200 cases, variables with statistically significant skewness or kurtosis typically do not deviate enough from normality to influence the analysis (Tabachnick and Fidell, 2000; White, 2008). Thus, if the sample is large enough, it is better to check the shape of the distribution, instead of using formal inference tests. Considering that the “standard errors for skewness and kurtosis decrease with large *N*, the null hypothesis will likely be rejected with significant cases when there are only minor deviations from normality” (Tabachnick and Fidell, 2000, p. 74). To examine the shape of the variable distributions, we checked the frequency histograms with superimposed normal distribution and expected standard probability plots. By assessing the plots, we concluded that all variables were considered normally distributed and maintained for examination using CFA.

We used structural equation modeling (SEM) to test the hypothesized structural relationships (H1 and H2) by examining regression coefficients among the constructs of interest for the hikers surveyed on Mt. Bukhan Dullegil. In addition, to test the mediating effect of the two dimensions of place attachment (i.e., place identity and place dependence) on the hypothesized relationship, we used the bootstrap method (Liu et al., 2020) to measure mediating effects (H3). The hypothesized model was assessed using the following goodness-of-fit indices: root mean square error of approximation (RMSEA under 0.10; MacCallum et al., 1996, Savalei, 2018), comparative fit indices (CFI greater than 0.95; Hu and Bentler, 1998, Savalei, 2018), the goodness of fit index (GFI greater than 0.90; Joreskog and Sorbom, 1986, Sahoo, 2019), and non-normed fit indices (NNFI greater than 0.90; Hu and Bentler, 1998, Hoe, 2008). All analyses were conducted using LISREL 8.7 and Mplus 7.0.

## 3 Results

### 3.1 Sociodemographic attributes of respondents

Our sample included a relatively diverse sample of outdoor recreationists. As shown in Table 3, about half of the 403 respondents were female (51.3%). The respondents’ mean age was 47.3 years, ranging from 17.0 to 84.0 (SD = 15.6). Their most frequent categories of monthly income were “below $1,800” (32.0%) and “$1,800 to $2,799” (22.1%). About half of the respondents were college graduates (53.7%). Most of them had visited the trails before (70.3%), while 29.7% were first-time visitors.

**Table 3:** Socio-demographic characteristics of survey respondents at Mt. Bukhan National Park, South Korea (*n* = 403).

		%
Gender	Male	48.7
	Female	51.3
Age	Ages (*M*, SD)	47.3 (15.7)
Education	High school graduate	37.9
	College graduate	53.7
	Graduate degrees	8.4
Monthly income	Less than $1,800	32.0
	$1,800 ~ $2,799	22.1
	$2,800 ~ $3,799	21.6
	$3,800 ~ $4,799	8.1
	More than $4,800	16.2
Previous visits	Yes	70.3

### 3.2 Measurement model

The measurement model was tested in our first step of analysis. The modification indices from the initial analysis suggested that fit would be improved by covarying error terms for item 2 with 1 and item 9 with 3. After reviewing the original scale for these items, we affirmed the decision to modify the model slightly based on similarities between items. For example, item 2 read “I enjoy natural environments” and item 1 read “I am interested in ecological preservation in Mt. Bukhan N. P.” Both items belonged to the same dimension (environmental sensitivity) and captured the affective aspect of an environmental attitude. Also, item 9 read “Visiting Mt. Bukhan N. P. has a deep meaning for me” and item 3 read “I appreciate the natural environment of Mt. Bukhan N. P.” Both items measured the perceived meaning of Mt. Bukhan N. P. and captured the value of visiting Mt. Bukhan. After allowing covariations between the error terms of those items, the final measurement model showed an acceptable fit (*χ*^2^ = 1092.168, df = 204, RMSEA = 0.060, NNFI = 0.953, CFI = 0.958; GFI = 0.894) (see Table 2). All other measurement model metrics were also satisfactory. Cronbach’s alpha coefficients for subscales were all greater than 0.80. Factor loadings ranged from 0.690 to 0.924. The composite reliability (CR) of all dimensions ranged from 0.840 to 0.935, showing good reliability across the constructs, and the AVE extracted was above 0.50 (Table 2).

### 3.3 Structural model

After establishing a valid measurement model, we tested the structural model (see Figure 1). We hypothesized that the two environmental attitude variables (i.e., environmental knowledge and sensitivity) would positively predict both dimensions of place attachment (i.e., place identity and place dependence). We hypothesized that these place attachment dimensions would, in turn, positively influence two types of environmental behaviors (i.e., off-site and on-site PEBs). The final structural model indicated a satisfactory model fit (*χ^2^* = 547.403, df = 195, RMSEA = 0.064, NNFI = 0.980, CFI = 0.983; GFI = 0.914).

**Figure 3:**
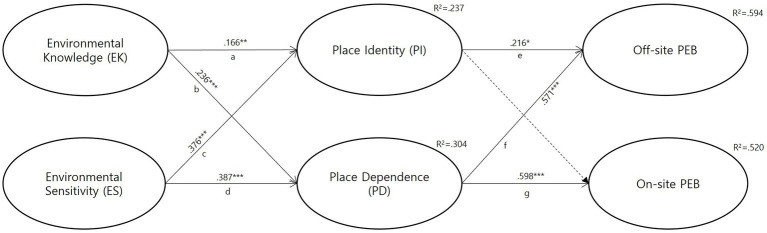
Structural equation model results depicting relationships among multiple dimensions of environmental attitudes, place attachment, and pro-environmental behavior for visitors to Mt. Bukhan National Park, South Korea (*n* = 403). The dotted line indicates insignificant relationship. Direct effects: there is no significant direct effects from EK/KS to PI/PD and from PI/PD to Off/On-site PEB. Indirect effects: a + e = 0.083**, b + f = 0.209***, b + g = 0.238***, c + e = 0.037*, d + f = 0.116**, d + g = 0.131**.

Table 4 and Figure 3 show the results of the structural equation model (SEM), including links between environmental attitudes, place attachment, and PEB. First, environmental knowledge positively influenced place identity (H1-1; *β* = 0.166, *t* = 2.627, *p* < 0.01) and place dependence (H1-2; *β* = 0.236, *t* = 3.877, *p* < 0.001). Second, environmental sensitivity positively influenced both place identity (H1-3; *β* = 0.376, *t* = 5.845, *p* < 0.001) and place dependence (H1-4; *β* = 0.387, *t* = 6.306, *p* < 0.001). Environmental knowledge and environmental sensitivity accounted for 23.7% of the variance of place identity and 30.4% of the variance of place dependence. Thus, all components of H1 were supported by the analysis. Third, place identity positively influenced off-site PEB (H2-1; *β* = 0.216, *t* = 2.059, *p* < 0.05) but did not influence on-site PEB (H2-2; *β* = 0.135, *t* = 1.143, *p* > 0.05). Lastly, place dependence positively affected both off-site PEB (H2-3; *β* = 0.571, *t* = 5.518, *p* < 0.001) and on-site PEB (H2-4; *β* = 0.598, *t* = 4.786, *p* < 0.001). Therefore, H2 was partially supported by the analysis. Place attachment dimensions accounted for 59.4% of the variance in off-site PEB and 52.0% of the variance in on-site PEB, demonstrating high levels of predictive power.

**Table 4:** Beta coefficients for the structural model examining links between environmental attitudes, place attachment, and pro-environmental behavior reported by visitors to Mt. Bukhan National Park, Korea (*n* = 403).

Hypothesis	Predictor	Dependent variable	*β*	*t*-value	*R* ^2^
H1-1	Environmental knowledge	Place identity	0.166^**^	2.627	0.237
H1-3	Environmental sensitivity		0.376^***^	5.845	
H1-2	Environmental knowledge	Place dependence	0.236^***^	3.877	0.304
H1-4	Environmental sensitivity		0.387^***^	6.306	
H2-1	Place identity	Off-site PEB	0.216^*^	2.059	0.594
H2-3	Place dependence		0.571^***^	5.518	
H2-2	Place identity	On-site PEB	0.135	1.143	0.520
H2-4	Place dependence		0.598^***^	4.786	

^*^p < 0.05, ^**^p < 0.01, ^***^p < 0.001.

We further analyzed the indirect effects to examine whether place attachment was a significant mediator of the relationship between the two types of environmental attitudes and off-site and on-site PEB. Specifically, bootstrapping results provided support for the causal relationships among the variables (see Table 5). None of the confidence intervals for the indirect effects included zero, indicating that there were significant indirect effects for all six paths in the hypothesized model. These analyses empirically demonstrated that environmental knowledge had a positive indirect effect on off-site PEB through place identity (path 1: indirect effect = 0.083, 95% CI = [0.032, 0.163], *p* < 0.01) and place dependence (path 2: indirect effect =0.209, 95% CI = [0.159, 0.330], *p* < 0.001). In addition, the indirect effects of environmental knowledge on on-site PEB were positively mediated by place dependence (path 3: indirect effect = 0.238, 95% CI = [0.163 0.325], *p* < 0.001). Environmental sensitivity also had a positive indirect effect on off-site PEB via place identity (path 4: indirect effect = 0.037, 95% CI = [0.002, 0.076], *p* < 0.05) and place dependence (path 5: indirect effect =0.116, 95% CI = [0.049, 0.194], *p* < 0.01). Lastly, the indirect effects of environmental sensitivity on on-site PEB were positively mediated by place dependence (path 6: indirect effect =0.131, 95% CI = [0.052 0.190], *p* < 0.001). Therefore, H3 was supported by the analysis.

**Table 5:** Summary of indirect effects of place identity and place dependence on the relationship between environmental attitudes and pro-environmental behaviors using bootstrapped 95% confidence intervals (lower and upper bounds).

Path	Indirect effect	Direct effect	S.E.	*Z*	95% C.I.	Total effect
Path 1: EK → PI → Off	0.083^**^	0.060	0.028	2.936	(0.032, 0.163)	0.143
Path 2: EK → PD → Off	0.209^***^		0.042	4.935	(0.159, 0.330)	0.269
Path 3: EK → PD → On	0.238^***^	0.065	0.045	5.351	(0.163, 0.325)	0.303
Path 4: ES → PI → Off	0.037^*^	0.088	0.018	2.067	(0.002, 0.076)	0.125
Path 5: ES → PD → Off	0.116^**^		0.037	3.137	(0.049, 0.194)	0.204
Path 6: ES → PD → On	0.131^**^	0.084	0.040	3.285	(0.052, 0.190)	0.215

^*^p < 0.05, ^**^p < 0.01, ^***^p < 0.001.

EK, environmental knowledge; ES, environmental sensitivity; PI, place identity; PD, place dependence; Off, off-site PEB; On, on-site PEB.

## 4 Discussion

Results of our study revealed that, for hikers at Mt. Bukhan National Park in South Korea, place attachment—and place dependence, specifically—had a powerful mediating influence on the relationship between pro-environmental attitudes and behavior. Furthermore, our study showed that both cognitive and affective aspects of environmental attitudes—and particularly affective components—influenced place attachment and indirectly impacted PEB (Figure 2).

Affective and cognitive components of environmental attitudes appeared to influence place attachment to different degrees and in different ways. For instance, we found that environmental sensitivity had a stronger effect than environmental knowledge on both place identity and place dependence. In other words, affection (environmental sensitivity) was relatively more important than cognition (environmental knowledge) when considering attachment to an outdoor recreation site. Although limited research has investigated the impact of environmental sensitivity on place attachment, many studies have revealed the importance of emotions and affective connections to place (Duff, 2010; Brehm et al., 2013). For example, Yeh et al. (2012) reported that cultural tourists’ emotional response to their destination contributed to the formation of place attachment. Additionally, Thailand tourists’ emotions were significant determinants of place attachment (Hosany et al., 2017). Furthermore, Kastenholz et al. (2020) revealed how individuals’ positive emotions toward a certain place positively influenced place attachment. Consistent with existing literature, our findings show that an emotional response to the natural environment, recognized as environmental sensitivity, may be more important than environmental knowledge and awareness when it comes to forging connections to places such as Mt. Bukhan Dullegil.

We also found significant associations between place attachment and the two types of PEB we examined: on-site and off-site. However, the mediating effect of place attachment varied in each case. Place identity only significantly affected off-site PEB, while place dependence influenced on-site and off-site PEBs. Previous studies have shown how PEB can be significantly influenced by identification with a place. For example, Carrus et al. (2005) found that local residents’ place identity positively affected the intention to protect two parks on the island of Sardinia and Gravina in Puglia, Italy. Likewise, Bonaiuto et al. (2008) studied local community residents living near the Gennargentu National Park in Italy and reported that the stronger their place identity was, the more readily cooperative they were in environmental protection behaviors. Vaske and Kobrin (2001) found that youth who participated in local natural resource work programs in Colorado and reported stronger place identity with outdoor recreation sites were more likely to engage in PEB. These relationships might be explained by a strong link between the formation of place identity, which often manifests as connection to nature or nature-relatedness in outdoor recreation contexts (Groulx et al., 2016), and broader environmental identities that influence PEBs in other aspects of life (Mackay and Schmitt, 2019). Individuals who identify with a place, and experience those connections, may be more inclined to engage in PEBs across many conservation contexts (Halpenny, 2010; Ramkissoon et al., 2013).

However, as previous suggests (Stedman, 2003), functional dependence on place could be even more important than identity for predicting participation in both off-site and on-site PEB. Although off-site or general environmental behavior (e.g., donations, advocacy) have been heavily emphasized in previous research (Larson et al., 2015), few studies have explicitly examined links between place attachment and on-site stewardship behaviors that directly affect resources in a particular park or outdoor recreation destination (Walker and Chapman, 2003). However, some research suggests that place attachment may have a particularly powerful influence on these site-specific actions (Larson et al., 2018a, 2018b). Our findings suggest that, in this on-site context, place dependence can play a more important role than place identity. There are various studies detailing the role of place dependence in outdoor recreation. Vaske and Kobrin (2001) reported that the place dependence of youth participants in natural resource education programs indirectly affected general (e.g., talking with others about environmental problems) and specific (e.g., joining in community clean-up efforts) environmental behaviors. Kyle G. et al. (2004) found that people with higher place dependence show more interest in the maintenance of quality nature-based recreational settings. Hence, recreationists who are attached to a place because it helps them achieve their recreational goals are more likely to engage in actions that protect that place (Halpenny, 2010; Ramkissoon et al., 2013). For instance, our respondents who considered Mt. Bukhan Dullegil the best hiking trail compared to similar alternatives (i.e., those with high place dependence) were more likely to exhibit both on-site and off-site behaviors that would protect and enhance the condition of the trail and the park as a whole. Consequently, an emphasis on building and maintaining place dependence, whether or through enhanced environmental knowledge or sensitivity or other functional mechanisms, could be a viable approach for managers hoping to encourage PEBs within and beyond the boundaries of outdoor recreation settings.

### 4.1 Limitations and future research

This study has several limitations. First, this study collected the data from one national park in South Korea that is located in the vicinity of the Seoul metropolitan area. Research suggests that place attachment varies across the recreation opportunity spectrum (Wynveen et al., 2020), and different recreation contexts would likely yield different levels of visitor attitudes and attachment. Additionally, since we operationalized environmental attitude as a two-dimensional construct, investigating other components of environmental attitudes in more diverse outdoor recreation contexts could be helpful to estimate the generalizability of current study findings. Second, to deepen our understanding in the predictors of PEBs, other factors not incorporated in the current study, such as social norms (Farrow et al., 2017), perceived behavioral control (Alshurideh et al., 2019), and cultural and symbolic interpretation/understanding of nature (Cordano et al., 2010), could be included in future models exploring the relationship between pro-environmental attitudes and behaviors. Third, future studies that intend to examine the role of place to predict PEBs could differentiate place meanings (see Brehm et al., 2013; Larson et al., 2018a) and place attachment (Raymond et al., 2010), considering both the level of attachment that exists and the diverse mechanism (environmental, social, etc.) through which those attachments develop. Fourth, specific questions about other types of behaviors that might occur within either the on-site context, such as Leave No Trace practices (Lawhon et al., 2017), or off-site practices, such as social environmentalism (Larson et al., 2015), could shed more light on PEB engagement patterns.

### 4.2 Management implications

This study has several implications for nature-based recreation and environmental research and practice. First, our finding that environmental knowledge positively predicted place attachment underscores the importance of education and interpretation in park settings. By providing information about the natural environment through various mediums (websites, flyers, guided tours, etc.) that increase visitors’ knowledge and awareness of key resources and recreation impacts, managers can also increase the affective connection to a recreation site (Hwang et al., 2005; Lawhon et al., 2017). Second, managers should also consider visitors’ emotional and affective responses to the environmental conditions at places such as Mt. Bukhan National Park because environmental sensitivity is associated with both place identity and place dependence. As other studies have shown, efforts to build connection to nature and create emotionally meaningful experiences for visitors to nature-based tourism destinations are likely to foster PEB (Folmer et al., 2013; Cajiao et al., 2022). Third, park managers hoping to achieve stewardship goals should seek ways to cultivate and strengthen visitors’ attachment to place. As place attachment can be fostered through additional time spent in a recreational setting (Smaldone, 2007), it is important to encourage return trips from first-time visitors, providing more opportunities for attachment to develop (Ramkissoon et al., 2018). Moreover, managers might pursue innovative ways of attracting new visitors to parks, such as outdoor educational programs or unique initiatives that draw in new users (e.g., the First Day Hike program on New Year’s Day in U.S. state parks, Wilcer et al., 2018). Collectively, these approaches can be used to facilitate positive visitor experiences while achieving sustainable management goals at popular destinations such as Mt. Bukhan, South Korea, and other heavily visited parks and protected areas worldwide.

## 5 Conclusion

This study built on previous work examining connections between environmental attitudes and PEBs by exploring different dimensions of PEB (onsite vs. offsite), investigating a potential mediating factor (i.e., place attachment), and collecting data in a novel research context (an Asian national park). Although both cognition (environmental knowledge) and affection (environmental sensitivity) increased outdoor recreationists’ place attachment to the national park, the affective aspect of environmental attitude was the stronger predictor of place attachment. Affective dimensions should therefore be considered in future research on place attachment and PEB. Whereas place identity was linked to off-site PEBs, place dependence was linked to both onsite and offsite PEBs. This finding suggests that, for managers hoping to inspire sustainable recreation behaviors among visitors, functional dependence on a destination may be key. Ultimately, our results indicate that managers hoping to promote PEB and responsible recreation at their sites should focus on fostering affective bonds between outdoor recreationist and the parks they visit.

## Data availability statement

The raw data supporting the conclusions of this article will be made available by the authors, without undue reservation.

## Ethics statement

The studies involving humans were approved by Kyung Hee University Global Campus Institutional Review Board. The studies were conducted in accordance with the local legislation and institutional requirements. The ethics committee/institutional review board waived the requirement of written informed consent for participation from the participants or the participants’ legal guardians/next of kin because the survey presents respondents with minimal potential risk (equivalent to a risk of daily lives).

## Author contributions

JY: Conceptualization, Formal analysis, Methodology, Writing – original draft, Writing – review & editing. KL: Supervision, Writing – review & editing. LL: Methodology, Writing – review & editing.
